# Fixing Flexner: Disrupting and Rebuilding Academic Medicine for Women of Color to Lead

**DOI:** 10.2196/47773

**Published:** 2023-05-10

**Authors:** Chelsea Dorsey, Vineet M Arora, Keme Carter

**Affiliations:** 1 Department of Surgery University of Chicago Medicine Chicago, IL United States; 2 Pritzker School of Medicine University of Chicago Chicago, IL United States; 3 Department of Medicine University of Chicago Medicine Chicago, IL United States

**Keywords:** Flexner Report, diversity, leadership, minority tax, Flexner, color, diverse, medical education, academia, women, minority, academic

## Abstract

In an effort to address the lack of compositional diversity seen in academic leadership, our generation has an opportunity to rebuild academic medicine in a way that welcomes, values, and supports the development and success of women of color.

## Introduction

Over a century has passed since the Flexner Report was published and prescribed standardization of the learning environment in medical education. The report, however, has been widely and appropriately criticized for recommending standards that led to the closure of most historically Black medical schools and the reversion to “male-only” admissions practices [1]. The structure and content of present-day US medical education as well as the persistent dearth of physician workforce diversity are evidence of the report’s enduring impact. Flexner’s recommendations effectively laid a foundation of structural racism, sexism, and misogynoir that, for generations, has limited access and opportunities within academic medicine for many. Without collective recognition of this foundation on which the house of medicine is built and deliberate effort to dismantle it, other forms of oppression including misogynistic-structured invisibility, lack of recognition, and pervasive stereotyping have emerged and gone unchecked, ultimately thwarting the progress of Black women and other women of color in academic medicine [2].

Despite increased attention to workforce diversity, particularly over the last 3 years, there has been negligible advancement for women of color in academic medicine. When one sharpens their focus to the highest echelons of leadership in academia, the unhurried pace is even more alarming with nonexistent representation in some areas. According to the Association of American Medical Colleges in 2022, 77% of department chair positions were held by men with three-quarters of those men identifying as White [3]. Cumulatively, women of color (all non-White groups) represent only 5% of department chairs. Black women, more specifically, only constitute 1.5% of chairs with a paucity seen particularly in basic science departments [3]. While these statistics undoubtedly have historical roots in systems of oppression, an understanding of how the status quo is actively maintained and the solutions to enact real change are urgently needed.

## The Problem

Though some may argue this simply represents “leaks” in a pathway to leadership, a more realistic interpretation is that in academia’s current state, these pathways are largely nonexistent for women of color. Furthermore, the isolation and hostility experienced by Black women and other women of color in academic medicine at every rank are, in the best case, discouraging yet potentially surmountable but more often outright destructive—devastating an individual’s career, self-worth, and dignity [4,5]. These and other barriers comprehensively described in existing literature have facilitated the loss of talent through attrition and reinforced an academic medicine structure with a scarcity of leaders who identify as women of color [4,5].

## The Solutions

### Overview

In academic medicine, the existing distribution of power and data reflecting compositional diversity at the highest ranks of leadership are certainly discouraging and overwhelming—but let them serve as a call to action. Our generation has an opportunity to disrupt the status quo, change course, and rebuild academic medicine in a way that welcomes, values, and supports the development and success of diverse leaders. To do this, we recommend focusing on ways that we can recognize the minority tax as a responsibility disparity experienced by minoritized faculty and assign value to diversity, equity, and inclusion (DEI) work; encourage, adequately support, and provide opportunities for women of color to lead in *all* spaces; and diversify and in some cases build from the ground up the pathways to the highest leadership positions in academic medicine.

### Recognize the Minority Tax and Assign Value to Diversity, Equity, and Inclusion Work

At the top of the action items is the need to take an inventory of how we recognize and consequently reward women of color who engage in academic activities that are widely undervalued such as DEI efforts; more clinical time to underserved patients; mentorship; and local community engagement. Minority faculty often feel an obligation to and sense of mission in work that advocates for and represents the interests of the communities that they represent. However, despite this work showing measurable and far-reaching impact with institutions enjoying the fruits of minority faculty labor, protected time is often not granted and academic promotion processes often fail to provide a framework for these accomplishments to be incorporated into the discussion. Furthermore, because academic rank primarily influences salary, a flawed and biased promotion process reinforces existing disparities in compensation [6]. Although in recent years many have advocated for promotion process reform, with some institutions taking action, far too often the work of women of color faculty is still passed over or even shunned as qualifiers for promotion—an unacceptable reality in 2023.

In some ways, the difficulty for women of color in converting these tasks into academic currency (and ultimately promotion) should not be surprising. The reality is that much of the process is not informed by theory or scholarship but rather guided by traditions and policies initially created decades ago by and for predominantly White men [7]. Though our generation should not be held solely responsible for guidelines, procedures, and programs that inherently disadvantage women of color, there must be accountability for a profession that insists on maintaining those processes without sincere attempts at reform. Academic promotions must move from an antiquated highly opaque process that is overly reliant on faculty self-agency to one that is transparent and places explicit value on impactful but currently marginalized activities. Beyond promotion, women of color should be given both protected time and financial compensation for their “taxed” activities.

### Encourage, Adequately Support, and Provide Opportunities for Women of Color to Lead

If not addressed, the lack of compositional diversity in leadership will ultimately influence our ability as a profession to adequately meet the health needs of the diverse communities we serve. In academia, there must be a collective and unified effort to support women of color to lead. This must be done in all ways and in all spaces—through intentional mentorship, sponsorship, and coaching by senior leaders [8]. Existing senior leaders must recognize and accept their key role as upstanders and integral parts of solutions to increase compositional diversity in leadership; they must take the time to get to know their faculty, understand their strengths, provide or ensure access to coaching so that gaps in leadership skills and knowledge are filled, and sponsor women of color for leadership opportunities in keeping with *their own* career goals. There is a tendency, whether intentional or unconscious, to assume that women of color are best suited to effectively contribute to the DEI space, when in reality there is a ubiquitous need for their leadership in all corners of academia. The “glass cliff” phenomenon must also be avoided, whereby women of color break through the proverbial glass ceiling but are positioned to do so in times of crisis, increasing their likelihood of failure [9]. Unfortunately, this penchant for typecasting women of color and asking them to lead at times of upheaval can have deleterious effects on one’s career that at times can be irreversible [10]. Support structures for women of color to lead effectively and successfully must be developed and enhanced to include adequate compensation, administrative resources, and advising.

### Diversify the Pathways to Senior Leadership in Academic Medicine

Lastly, the pathways to leadership in academia must be expanded. Recent studies have confirmed that the most common pathway to higher-level leadership positions such as department chair typically includes experience in administrative roles focused on strategy, finance, or policy [11,12]. Furthermore, the path to institutional deanship typically includes tenure as a department chair [11]. The existing literature has described women and minority faculty as more likely to be involved in education and DEI efforts, respectively [13,14]. As such, the propensity for women of color to populate these roles inherently puts them at a disadvantage as it relates to advancing to the top leadership positions in academia. What is interesting to note is that these roles are often perceived as positions devoid of strategy, finance, or policy—when, in fact, nothing could be further from the truth. DEI efforts within a large clinical department, for example, when operationalized effectively require leaders to be change agents who can create and incorporate new policies, work alongside other departmental leaders to formulate overarching strategic priorities, and often oversee a large budget. In many ways, successful DEI leaders are well positioned to transition to larger roles such as department chair given the crosscutting nature of the role, which requires knowledge and familiarity with all facets of a department including quality, compensation, faculty development/promotion, and education. The responsibility of faculty leadership appointments often falls on the shoulders of existing faculty leaders who must recognize the broad and transferable skill sets DEI and medical education leaders acquire that would bring value to the highest levels of institutional leadership.

## Conclusions

The Flexner Report has left deep wounds in academia felt profoundly by exceptional Black women and other women of color whose talents are either wasted or never brought to fruition. Through institutional self-reflection and strategic investments in the 3 solution areas discussed, medical institutions across the country can forge a path forward where women of color can not only find a home in academia but also have the overdue opportunity to lead the way.

## Abbreviations

DEI: diversity, equity, and inclusion
